# Mesoporous polydopamine nanoparticles carrying peptide RL-QN15 show potential for skin wound therapy

**DOI:** 10.1186/s12951-021-01051-8

**Published:** 2021-10-09

**Authors:** Pan Qin, Yi Meng, Ying Yang, Xinyu Gou, Naixin Liu, Saige Yin, Yan Hu, Huiling Sun, Zhe Fu, Yinglei Wang, Xiaojie Li, Jing Tang, Ying Wang, Ziwei Deng, Xinwang Yang

**Affiliations:** 1 Department of Anatomy and Histology and Embryology, Faculty of Basic Medical Science, Kunming Medical University, Kunming, Yunnan 650500 China; 2 Key Laboratory of Applied Surface and Colloid Chemistry, National Ministry of Education, Shaanxi Key Laboratory for Advanced Energy Devices, Shaanxi Engineering Lab for Advanced Energy Technology, School of Materials Science and Engineering, Shaanxi Normal University, Xi’an, Shaanxi 710119 China; 3 Key Laboratory of Chemistry in Ethnic Medicine Resource, State Ethnic Affairs Commission and Ministry of Education, School of Ethno-Medicine and Ethno-Pharmacy, Yunnan Minzu University, Kunming, Yunnan 650504 China; 4 Department of Biochemistry and Molecular Biology, Faculty of Basic Medical Science, Kunming Medical University, Kunming, Yunnan 650500 China; 5 Department of Endocrinology and Metabolism, Second People’s Hospital of Yunnan Province and Affiliated Hospital of Yunnan University, Kunming, Yunnan 650021 China

**Keywords:** Wound healing, Mesoporous polydopamine, RL-QN15, Nanoparticles

## Abstract

**Background:**

Skin wound healing remains a considerable clinical challenge, thus stressing the urgent need for the development of new interventions to promote repair. Recent researches indicate that both peptides and nanoparticles may be potential therapies for the treatment of skin wounds.

**Methods:**

In the current study, the mesoporous polydopamine (MPDA) nanoparticles were prepared and the peptide RL-QN15 that was previously identified from amphibian skin secretions and exhibited significant potential as a novel prohealing agent was successfully loaded onto the MPDA nanoparticles, which was confirmed by results of analysis of scanning electron microscopy and fourier transform infrared spectroscopy. The encapsulation efficiency and sustained release rate of RL-QN15 from the nanocomposites were determined. The prohealing potency of nanocomposites were evaluated by full-thickness injured wounds in both mice and swine and burn wounds in mice.

**Results:**

Our results indicated that, compared with RL-QN15 alone, the prohealing potency of nanocomposites of MPDA and RL-QN15 in the full-thickness injured wounds and burn wounds in mice was increased by up to 50 times through the slow release of RL-QN15. Moreover, the load on the MPDA obviously increased the prohealing activities of RL-QN15 in full-thickness injured wounds in swine. In addition, the obvious increase in the prohealing potency of nanocomposites of MPDA and RL-QN15 was also proved by the results from histological analysis.

**Conclusions:**

Based on our knowledge, this is the first research to report that the load of MPDA nanoparticles could significantly increase the prohealing potency of peptide and hence highlighted the promising potential of MPDA nanoparticles-carrying peptide RL-QN15 for skin wound therapy.

**Graphic abstract:**

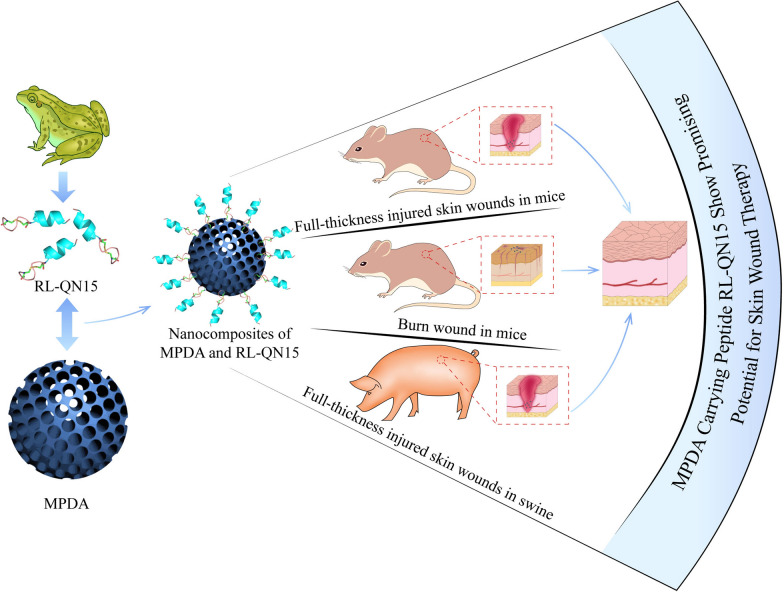

**Supplementary Information:**

The online version contains supplementary material available at 10.1186/s12951-021-01051-8.

## Introduction

Skin is exposed to many external and endogenous factors that can impact structure and function, making it easy to form wounds. Rapid repair after skin damage is an important precondition for protecting the internal organs from the external environment, which mainly consists of four stages: hemostasis, inflammatory response, cell proliferation and tissue reconstruction [1]. Physiological regulation of skin wound healing is a complex process that depends on interactions among many cell types and mediators in a highly sophisticated temporal sequence [2]. Therefore, it can be interrupted by many factors, resulting in non-healing chronic wounds or unhealing skin ulcer. An aging population coupled with escalating rates of diabetes and obesity continue to increase the prevalence of chronic wounds, leading to a sharp rise in medical costs [3]. Chronic wounds cause secondary infections, as well as water and electrolyte disturbance, infectious shock, multiple organ failure, and even death [4, 5]. Thus, the integrity of skin structure and function is really crucial for human health and survival.

While extensive research has been conducted to identify new methods for improving skin wound healing, it still fails to meet clinical needs. Traditional wound healing drugs can be classified into two main groups: small molecule compounds derived from plants and proteins typified by epidermal growth factors (EGFs). Although these drugs can promote wound healing, there are still drawbacks; for example, the former compounds are unstable with relatively low activity, and the latter proteins are expensive, require rigorous preservation conditions, and are liable to cause hyperplastic scars [6]. In addition, stem cells from various sources are considered to be an attractive alternative method for wound repair [7–9]. However, the poor viability of stem cells in wound beds characterized by a harsh inflammatory environment often decreases the therapeutic potential of the cells [10, 11]. Thus, the development of novel promising interventions for the treatment of chronic skin wounds are definitely urgently awaited.

At present, nanomaterials have provided additional therapeutic strategies for wound repair. The main types of nanomaterials used for wound treatment are represented by nanoparticles, nanocomposites, hydrogels and scaffolds. Nanoparticles in treating wounds are mainly due to their intrinsic characteristics that aid wound closure or as delivery vectors for therapeutic agents. In particular, nano‑drug delivery vectors can anchor bioactive molecules to an applied area, sustain drug release, and enhance the therapeutic efficacy of drugs, and are thus particularly relevant to the field of skin regeneration [12]. Mesoporous polydopamine (MPDA), as an excellent carrier featured by simple preparation protocol, strong adhesive properties, easy and straightforward functionalization, and biocompatibility, has the following preconditions: (1) The mesoporous structure of MPDA can be loaded into drug molecules; (2) MPDA contains many functional groups such as catechol, amine, and imine for chemical modification [13, 14]. In addition, MPDA nanoparticles were synthesized with uniform pores by a one-pot method without additional catalysts or toxic reagents, which has the advantages of simplicity, mildness, and environmental friendliness. However, the reports on the improvement of bioavailability fulfilled by MPDA nanoparticles still remain in their infancy. Previous report has revealed that the drug doxorubicin hydrochloride is encapsulated in the MPDA particles to kill the cancer cells and shows an unprecedentedly high payload [15]. However, the attempt to accelerate the regeneration of skin wounds afforded by the MPDA nanoparticles is still rarely reported.

In recent years, peptides have gradually attracted extensive interest due to their diverse functions and high activity, stability, and specificity [16–18]. Recent researches have shown that peptides from plants and animals have a wide range of bioactivities, e.g., antimicrobial, hyperuricemia-alleviating, pro-healing, and antioxidant activities [19–22]. We previously identified a short peptide (named RL-QN15, primary sequence ‘QNSYADLWCQFHYMC’) from the skin secretions of *Rana limnocharis*, which promoted the proliferation and migration of skin cells [23]. When at concentrations of nM levels, RL-QN15 significantly promote the healing of full-thickness injured wounds and diabetic skin chronic wounds in mice. However, we still hope that the ability of RL-QN15 to promote repair activity can be improved by some ways, so as to provide new strategies for the development of novel therapeutics for the treatment of wound healing which are still a harsh clinical challenge.

**Scheme 1:**
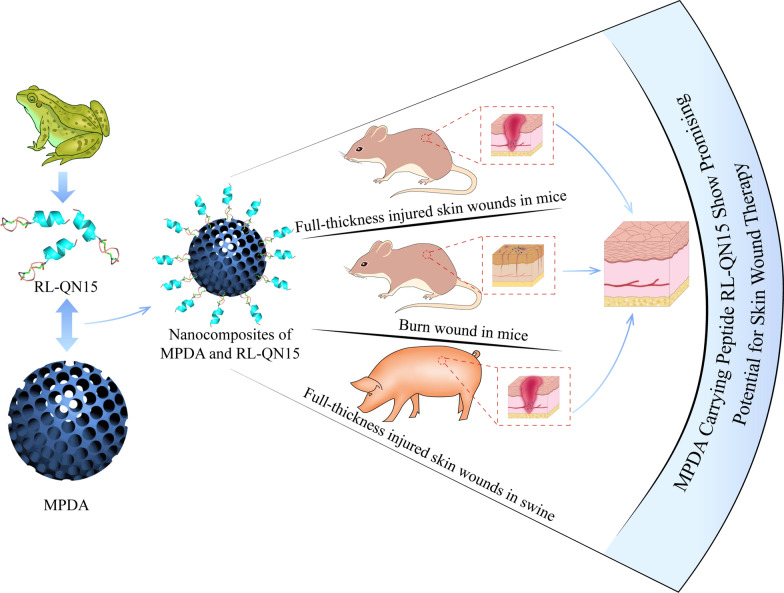
Peptide RL-QN15 exerts increase skin wound healing potency by the load of MPDA

In the current research, we successfully prepared the MPDA nanoparticles and constructed the nanocomposites of MPDA and RL-QN15. The prohealing potency of RL-QN15 in full-thickness injured skin wounds (mice and swine) and burns (mice) was significantly enhanced by the load of MPDA nanoparticles. The synthesis process and application in different wound types of nanocomposites of MPDA and RL-QN15 are shown in Scheme 1. At present, some studies have shown that MPDA nanoparticle is an excellent delivery carrier for drugs to inhibit tumor growth [24]. Based on our knowledge, this is the first research to report that the load of MPDA nanoparticles could significantly increase the prohealing potency of peptide and hence highlighted the nanocomposites of MPDA and RL-QN15 as a promising intervention for the acceleration of skin wounds.

## Experimental section

### Materials

All chemical reagents were of analytical grade and used as received without further purification. Dopamine hydrochloride (DA, 98%), triblock copolymer Pluronic F-127, and 1, 3, 5-trimethylbenzene (TMB) were purchased from Sigma-Aldrich (USA). Aqueous ammonia (NH_3_·H_2_O, 25.0–27.0%) and ethanol were purchased from Sino-Pharm Chemical Reagent Co., Ltd (China). Peptide with purity higher than 95% used in this research were commercially synthesized by Wuhan Bioyeargene Biotechnology Co. Ltd. (Wuhan, China).

### Ethics statement

All animal experimental procedures followed the Guide for the care and Use of Laboratory Animals and were approved by the animal ethics committee of Kunming Medical University, China (kmmu20211351). All surgeries were performed under anesthesia, and all efforts were made to minimize suffering.

### Synthesis of MPDA nanoparticles

The MPDA particles were synthesized by a one-pot synthesis method according to previously reported procedures [25]. Typically, 0.15 g of dopamine and 0.10 g of triblock copolymer Pluronic F-127 were first dissolved in a mixture of water (5 mL) and ethanol (5 mL) under stirring at room temperature. Then, 0.16 mL of TMB was introduced to the mixture. After ultrasonication for 2 min in a water bath, the mixture gradually formed an emulsion solution. Subsequently, 0.375 mL of ammonia solution was quickly added to the reaction mixture under stirring. After 2 h reaction under room temperature, the resultant mesoporous PDA particles were collected by centrifugation and washed with ethanol and water for three times. Template removal was performed by extraction, where the as-prepared PDA particles were treated in a mixed solvent of ethanol and acetone (v/v, 2/1) under ultrasonication for 30 min (three times). Finally, the obtained MPDA particles were re-dispersed in ultrapure water for further use.

### The preparation of nanocomposites of MPDA and RL-QN15

The MPDA nanoparticles were completely dispersed in PBS by an ultrasonic device (VCX750, Nanjing Xinchen Biotechnology Co., Ltd, China), then the peptide RL-QN15 were stirred with the dispersed MPDA nanoparticles at 4 °C for 12 h on a magnetic stirrer (Thermo Scientific, USA) to form nanocomposites of MPDA and RL-QN15.

### Characterization of MPDA

Field-emission scanning electron microscopy (FE-SEM, SU8020, HITACH, Japan) was used to investigate the surface morphologies and microstructures of MPDA particles. Transmission electron microscopy (TEM) images and mapping results were obtained using a Tecnai G2 F20 field emission transmission electron microscope (FEI, USA). Nitrogen adsorption-desorption isotherms and Brunauer-Emmett-Teller (BET) surface areas were measured with an ASAP 2460 Analyzer (Micromeritics Instruments, USA) at − 196 ℃.

### Characterization of nanocomposites of MPDA and RL-QN15

FE-SEM (FE-SEM, SU8220, HITACH, Japan) was used to investigate the surface morphologies and microstructures of nanocomposite of MPDA and RL-QN15. The spectral range of 4000 and 400 cm^− 1^ (mid-infrared) of MPDA and nanocomposites of MPDA + RL-QN15 were analyzed by using a Fourier transform infrared spectroscopy (FT-IR, NICOLET-IS10, Thermo Scientific, USA) in the attenuated total reflection sampling mode. X-ray photoelectron spectroscopy (XPS) measurement was performed on a Thermo Scientific K-Alpha X-ray photoelectron spectrometer (Thermo Scientific, USA) equipped with a monochromatized Al Ka X-ray source (1486.6 eV).

### Drug encapsulation efficiency

RL-QN15 was dissolved in PBS solution and its relative concentration was determined by spectrophotometer (GeneQuant 100, USA). Then 1 mg MPDA was dispersed in RL-QN15 solution and stirred at 4 ℃ for 24 h to form nanocomposites solution, which were then centrifuged (10, 000 rpm, 5 min, 4 ℃) at different time points, and the supernatant was taken to determine its concentration. The drug encapsulation efficiency of MPDA loaded RL-QN15 was calculated by the equation as follows:

Encapsulation efficiency (%) = [C_(0)_− C_(2, 4, 6, 10, 16, 24)_]/C_(0)_ × 100. where C_(0)_ represents the initial concentration, and C_(2, 4, 6, 10, 16, 24)_ represents the concentration in 2, 4, 6, 10, 16, 24 h, respectively.

### In-vitro drug release studies

The above successfully encapsulated nanocomposites of MPDA and RL-QN15 were re-suspended in PBS and Dulbecco’s modified Eagle’s medium (DMEM, BI, Israel). solution respectively and shook at speed of 80 rpm at 37 ℃. The release of peptide was analyzed by monitoring the content of RL-QN15 in the supernatant after centrifugation (10,000 rpm, 2 min) at different time intervals over a period of 24 h. The cumulative released RL-QN15 was calculated by the equation as follows:

Cumulative released of RL-QN15 (%) = C _(t)_/C _(0)_ × 100. Where C_(t)_ represents the concentration of RL-QN15 in the supernatant at different time points, and C_(0)_ represents the initial concentration of RL-QN15 in the nanocomposites.

### Effects of nanocomposites of MPDA and RL-QN15 on the healing of full-thickness dorsal skin wounds in mice

Adult Kunming male mice (n = 30, 20–24 g) form the same generation were purchased from Hunan SJA Laboratory Animal Co., Ltd. The mice were provided with essential food and water and the reverse of 12:12-h light/dark cycle. After five days of acclimatization, two identical round full-thickness cutaneous wounds (φ 10 mm) were created on the back of each mice, as previous research [22]. Mice were then randomly assigned into 4 groups, which were either treated with MPDA (20 µL, 0.2 mg/mL) + RL-QN15 (20 µL, 1 nM), RL-QN15 (20 µL, 1 nM), MPDA (20 µL, 0.2 mg/mL) or PBS (20 µL) once daily. Mice were kept in individual ventilated cages (FENGSHI, China) system with laboratory animal room of Kunming Medical University. For observing the wound healing process, the wounds were observed and captured by a digital camera at day 0, 2, 4, 6 and 8. Wound condition was documented, with ImageJ software (NIH, USA) employed to estimate wound area (percentage of residual wound area to initial area) and GraphPad Prism software used to quantify the rates of wound healing. Mouse skin wound tissues (n = 5) were isolated at days 4 and 8, and analyzed with Hematoxylin & Eosin (H&E) and light microscopy.

### Effects of nanocomposites of MPDA and RL-QN15 on the healing of burn wounds in mice

Adult Kunming male mice (n = 30, 20–24 g) were obtained from Hunan SJA Laboratory Animal Co., Ltd. Mice were provided with essential food and water and maintained under a reverse 12:12-h light/dark cycle. Mice were given burn skin wounds after 7 days of acclimation. As previous research, two burn wounds were formed on the back of each mouse [26]. Briefly, mice were anesthetized with 4% chloral hydrate (Solarbio, Beijing, China) at 10 mL/kg. Dorsal hair was shaved by an electric clipper and the skin was sterilized with 75% alcohol. A special metallic device containing a 10-mm diameter disc was used for inducing the experimental wound. The device was heated in boiling water and applied on the shaved dorsal area for 15 s. Mice were then randomly assigned into 4 groups, which were either treated with MPDA (20 µL, 0.2 mg/mL) + RL-QN15 (20 µL, 1 nM), RL-QN15 (20 µL, 1 nM), MPDA (20 µL, 0.2 mg/mL) or PBS (20 µL) twice daily. For observing the wound healing process, wounds were observed and captured by a digital camera at day 1, 4, 8, and 12. Wound areas (percentage of residual wound area to original wound area) were estimated from the photographs using ImageJ software (NIH, USA), in which the edges of the wounds were traced and the area of the pixels was calculated. Mouse skin wound tissues (n = 5) were isolated at days 8 and 12, and analyzed with H and E staining and light microscopy.

### Effects of nanocomposites of MPDA and RL-QN15 on the healing of acute full-thickness dorsal skin in swine

Bama swine (n = 2, 12–14 kg) were obtained from the Experimental Animal Center of Kunming Medical University. Swine were provided with essential food and water and the reverse of 12:12-h light/dark cycle. The swine were made full-thickness skin wound models after 7 days’ acclimation. Before the operation, animals were fasted for 12 h, but given access to water. Chlorpromazine (0.15 mL) was intramuscularly injected for sedation. After 5 min, the pigs received a gluteal intramuscular injection of 3% pentobarbital sodium (1 mL/kg). Hair was then shaved, and skin was washed with saline and sterilized with 75% alcohol. Using a permanent marker and a 30 × 30 mm template, wounds were drawn 30 mm apart on the dorsal skin to allow for adequate healing. A sterile No. 22 scalpel blade was used to excise the marked areas down to the fat layer. Finally, 12 wounds of the same size were formed on the back of each swine. The wounds were randomly assigned into 4 groups, which were topically treated with PBS, PDA (0.2 mg/mL), RL-QN15 (250 nM), or nanocomposites of MPDA (0.2 mg/mL) and RL-QN15 (250 nM) once a day. Wound condition was documented, with ImageJ employed to estimate wound area (percentage of residual wound area to initial area) and GraphPad Prism used to quantify wound healing. The tissues of wounds on the post-operative 28 day were further analyzed histologically by H&E staining.

### Histological analysis

All tissue samples isolated from animal were fixed in 4% paraformaldehyde for 24 h, and then dehydrated and hyalinized as per previous study [27]. For histological analysis, the center of the repair site was sliced into 5-µm sections followed by H&E and Masson trichrome staining. All slices were imaged by a Primovert microscope (Zeiss, Germany) and used to observe epidermal regeneration and granulation formation. For the evaluation of neo-epidermal thickness and granular thickness in acute skin wounds, five values were randomly measured in a field and mean values were calculated using ImageJ software (NIH, USA).

## Result and discussion

### Preparation and characterization of MPDA

Owing to their characteristics of surface functionalization, targeting, good degradability and biocompatibility, nanoparticles have been widely used in modern biomedicine as interventions of anti-tumor, antioxidation, drug delivery and tissue regeneration, et al. Recently, the application of nanoparticles in the field of wound healing has gradually attracted extensive attention, for example, the nanofibrous matrix based on biomimetic elastomeric peptide has been developed to overcome the multidrug-resistant bacterial and promote the skin regeneration [28], in addition, grape seed-inspired smart hydrogel scaffolds has also been reported to significantly accelerate the healing of wound [29]. MPDA nanoparticles shows unique characteristics featured by simple preparation protocol, strong adhesive properties, easy and straightforward functionalization, and biocompatibility, and thus has been widely used in the fields of biomedicine, sensing, catalysis, environment and energy [30]. However, for such an important nanoparticle, there are few reports on its application in accelerating the regeneration of skin wounds.

**Fig. 1:**
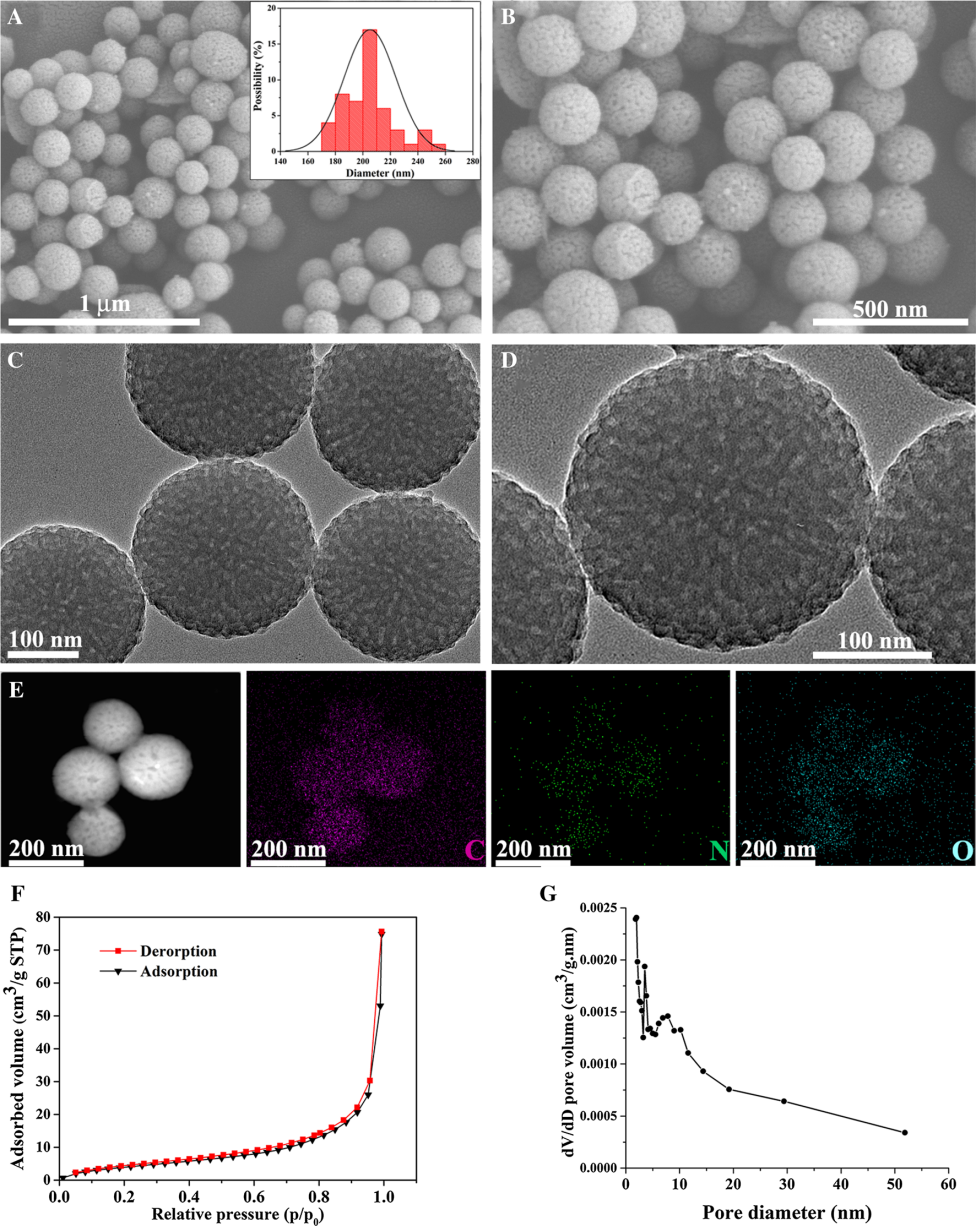
The characterization of MPDA. **A**, **B** FE-SEM images and (**C**, **D**) TEM images of MPDA particles. High magnification SEM images in **B** and TEM images in **D** showed typical MPDA particles. Inset in **A** showed corresponding size distribution histograms and theoretical fitting of MPDA particles. **E** Dark field TEM and energy-dispersive X-ray spectroscopy-based elemental mapping analysis of MPDA particles. Scale bars were indicated by lines in the figures

In the current research, MPDA nanoparticles were synthesized according to previously reported procedures [25]. As described in the “Experimental section”, MPDA nanoparticles were formed in an aqueous solution containing triblock copolymer Pluronic F-127 and 1, 3, 5-trimethylbenzene (TMB) as organic templates. Dopamine self-polymerized into PDA particles through an ammonia-catalyzed approach, and the resultant PDA particles were assembled on Pluronic F-127-stabilized TMB droplets via π–π stacking interactions. The PDA particles were finally obtained as the organic templates were removed. Both FE-SEM (Fig. 1A, B) and TEM images (Fig. 1C, D) confirmed that the resultant PDA particles showed well-defined spherical morphologies and mesoporous structures. Their average diameter and polydispersity were 205 nm and 3.9%, respectively (see inset in Fig. 1A). Close observation of the MPDA nanoparticles at higher magnification (Fig. 1B, D) revealed that the cylindrical open channels were exposed on the MPDA nanoparticle surfaces, in which some mesopores had diameters of 10 nm. The elemental mapping patterns revealed a uniform distribution of C, N, and O elements (Fig. 1E), further verifying the formation of MPDA nanoparticles. As shown in Fig. 1F, G, the nitrogen adsorption-desorption isotherms of MPDA demonstrated hysteresis loops of type IV, which is characteristic of mesoporous materials. The results revealed that the prepared MPDA nanoparticles had a surface area of 21.5475 m^2^/g, pore volume of 0.119326 cm^3^/g, and pore diameter of 21.3857 nm (Additional file 1: Table S1). Thus, due to their mesoporous structure, large surface area, and nano-sized spherical morphology, MPDA particles exhibit great potential as versatile platforms for drug delivery, diagnosis, and therapy [31].

### Characterization of nanocomposites of MPDA + RL-QN15

As a molecular tool, peptides have not only contributed substantially to clarify physiological processes in humans but also led to the discovery and development of novel therapeutics, hence it is widely recognized that peptide molecules have made indelible contributions to both basic scientific research and development of new drugs that are represented by insulin, exenatide, hirudin, captopril, et al. [32]. Amphibian skin secretions are rich in a variety of bioactive peptides, such as antimicrobial peptides, antioxidant peptides, bradykinins, neuromodulating peptides, and neurotoxins, et al. and thus have been considered as a treasure trove of the natural bioactive peptide [16, 33, 34]. In recent years, peptides with obvious potency of accelerating the healing of skin wounds have aroused significant attention and several prohealing peptides have been identified, including RL-QN15, cathelicidin-OA1 and cathelicidin-NV, Ot-WHP, tylotoin, OM-LV20, et al. [22, 35–39]. Our previous research have revealed that RL-QN15 contains an amino acid sequence of QNSYADLWCQFHYMC and an intramolecular disulfide bond located between C^9^ and C^15^, when at a relative low concentration of nM scale, more importantly, RL-QN15 significantly promote the healing of full-thickness injured wounds and diabetic skin chronic wounds in mice, and thus is considered as a promising prohealing agent [23]. However, we still hoped that the ability of RL-QN15 to promote repair activity can be improved by some ways, so as to provide new strategies for the development of novel therapeutics for the treatment of wound healing which are still a harsh clinical challenge. One of the available methods was to use the nanoparticles to enhance the prohealing potency of RL-QN15.

**Fig. 2:**
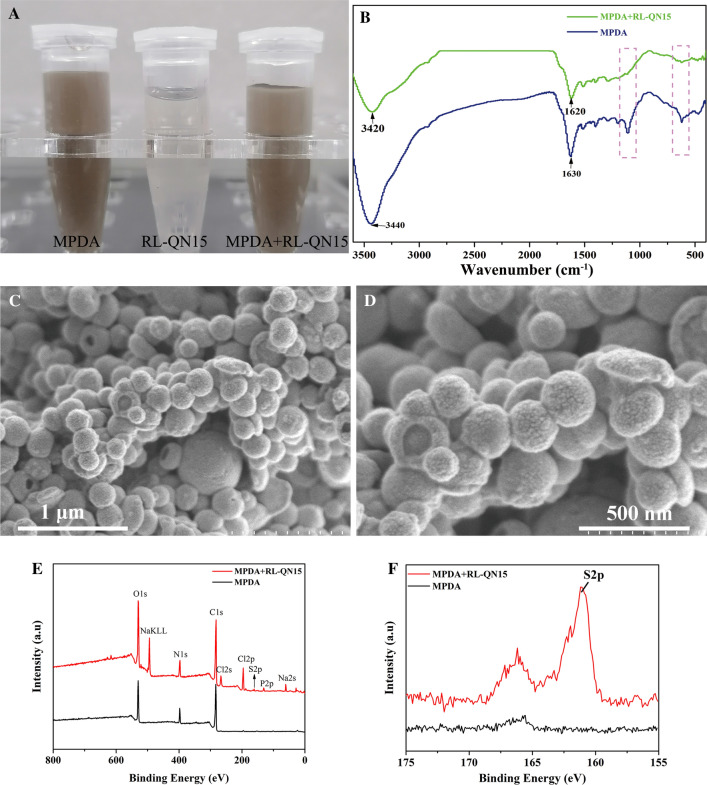
Characterization of nanocomposites of MPDA + RL-QN15. **A** The representative images of MPDA dispersed in PBS, RL-QN15 dissolved in PBS and nanocomposites of MPDA + RL-QN15 in PBS. **B** The FT-IR spectra of MPDA and nanocomposites of MPDA + RL-QN15. **C**, **D** SEM images of nanocomposites of MPDA + RL-QN15 at magnification of ×40,000 and 80,000, respectively. Scale bars were indicated by lines in the figures

In the current research, considering the characteristics of MPDA adhesion to each other, we first dispersed the prepared MPDA in PBS, as shown in Fig. 2A, the PBS solution of RL-QN15 is colorless, MPDA and MPDA + RL-QN15 nanocomposites are brown. Here, RL-QN15 with a molecular weight of only 1906 Da is completely soluble in water and PBS. Then, FT-IR analysis, which is useful in qualitative analysis as no two bioactive compounds have the same FT-IR spectra [40], was carried out to verify whether RL-QN15 was successfully loaded onto the MPDA shell. Figure 2B showed the characteristic peaks of MPDA and MPDA + RL-QN15 nanocomposites. The broad peak at 3440 cm^− 1^ in the PDA spectra was the characteristic adsorption of amine N-H and phenolic O-H stretching vibrations, and the band centered at 1 630 cm^− 1^ was assigned to the formation of an indole-related structure after the oxidation and self-polymerization of dopamine [41, 42]. Compared with MPDA, the wavenumber of the MPDA + RL-QN15 nanocomposites was lower at these two sites, which was attributed to the intermolecular hydrogen bond between MPDA and RL-QN15. In addition, there was a significant difference in the spectral fingerprint (1800 − 500 cm^−1^) between MPDA and MPDA + RL-QN15 nanocomposite. The characteristic absorption peaks of MPDA could be observed in the spectrum at the dotted frames, while the characteristic absorption peaks of MPDA/RL-QN15 nanocomposites were obviously weakened or even disappeared in the two places. These results suggested that RL-QN15 was present in MPDA nanoparticles. In addition, results of SEM also provided evidence of the successful formation of MPDA + RL-QN15 nanocomposite. Compared with the MPDA shell (Fig. 1A, B), the surface of the MPDA + RL-QN15 nanocomposites was rough and contained small particles (Fig. 2C, D). The diameter of MPDA and nanoparticles of MPDA + RL-QN15 shared similar diameter of about 200 nm. The X-ray photoelectron spectroscopy (XPS) experiments provided additional evidence for the successful loading of peptides onto MPDA nanoparticles. Our results showed that the characteristic binding energy peaks at 283, 398, and 530 eV are attributed to the C, N, O elements present in MPDA and MPDA + RL-QN15, and the nanocomposites have more binding energy peaks, with the peak at 161 eV due to the S element present in the Met and Cys of the peptide. RL-QN15 was successfully loaded into the mesoporous channel inside the MPDA NPs by sufficient stirring with the MPDA particles and hydrogen bonding of the catechol, amine, and imine groups on the nanoparticle surface.

In summary, we successfully prepared nanocomposite of MPDA + RL-QN15, and then we focused on the loading efficiency and release profile of the composites.

### In-vitro drug release and encapsulation efficiency of RL-QN15 loaded MPDA nanocomposites

**Fig. 3:**
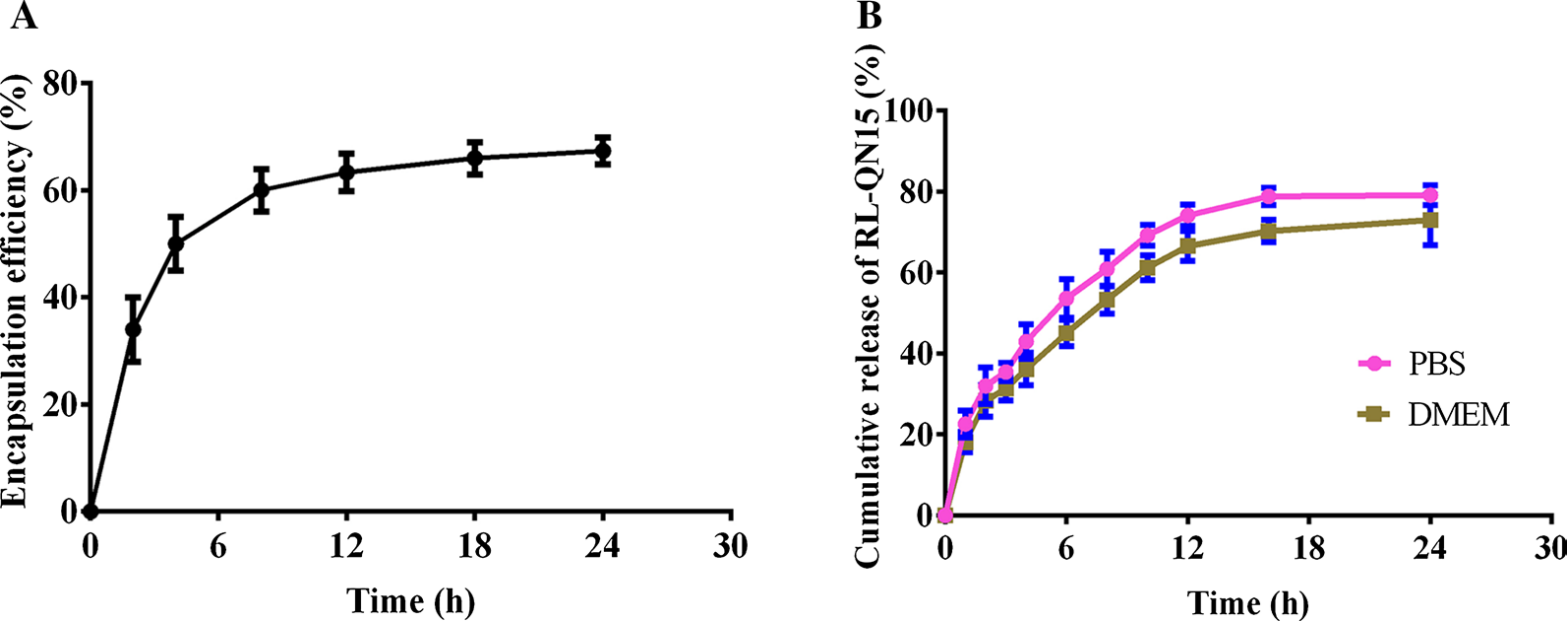
Loading efficiency and RL-QN15 release profile of MPDA/RL-QN15 nanocomposites. **A** The encapsulation efficiency of nanocomposites of MPDA and RL-QN15 at different time. **B** Release profiles of RL-QN15 from nanocomposites of MPDA and RL-QN15 in PBS solution and cell culture medium

The loading and releasing of RL-QN15 is based on the porous structure of MPDA microspheres. Here, based on the specific absorption peak of the peptide bond at 220nm, we made a standard curve of concentration vs. absorbance. As shown in Fig. 3A, the encapsulation rate of MPDA + RL-QN15 nanocomposites slowed down significantly after 6 h, and the maximum encapsulation was 67.31%. In addition, the slow release of RL-QN15 from MPDA microspheres has also been studied. As shown in Fig. 3B, RL-QN15 was continuously released from the microsphere during the period of 0–16 h, and the release rate was 79.12% at 24 h.

The MPDA microsphere nanoparticles have the characteristics of efficient loading and slow release of RL-QN15 and in the following experimental procedures, the main focus was to verify if the load of MPDA significantly enhanced the prohealing potency of RL-QN15.

### The load of MPDA significantly enhanced the prohealing potency of RL-QN15 on full-thickness injured wounds in mice

**Fig. 4:**
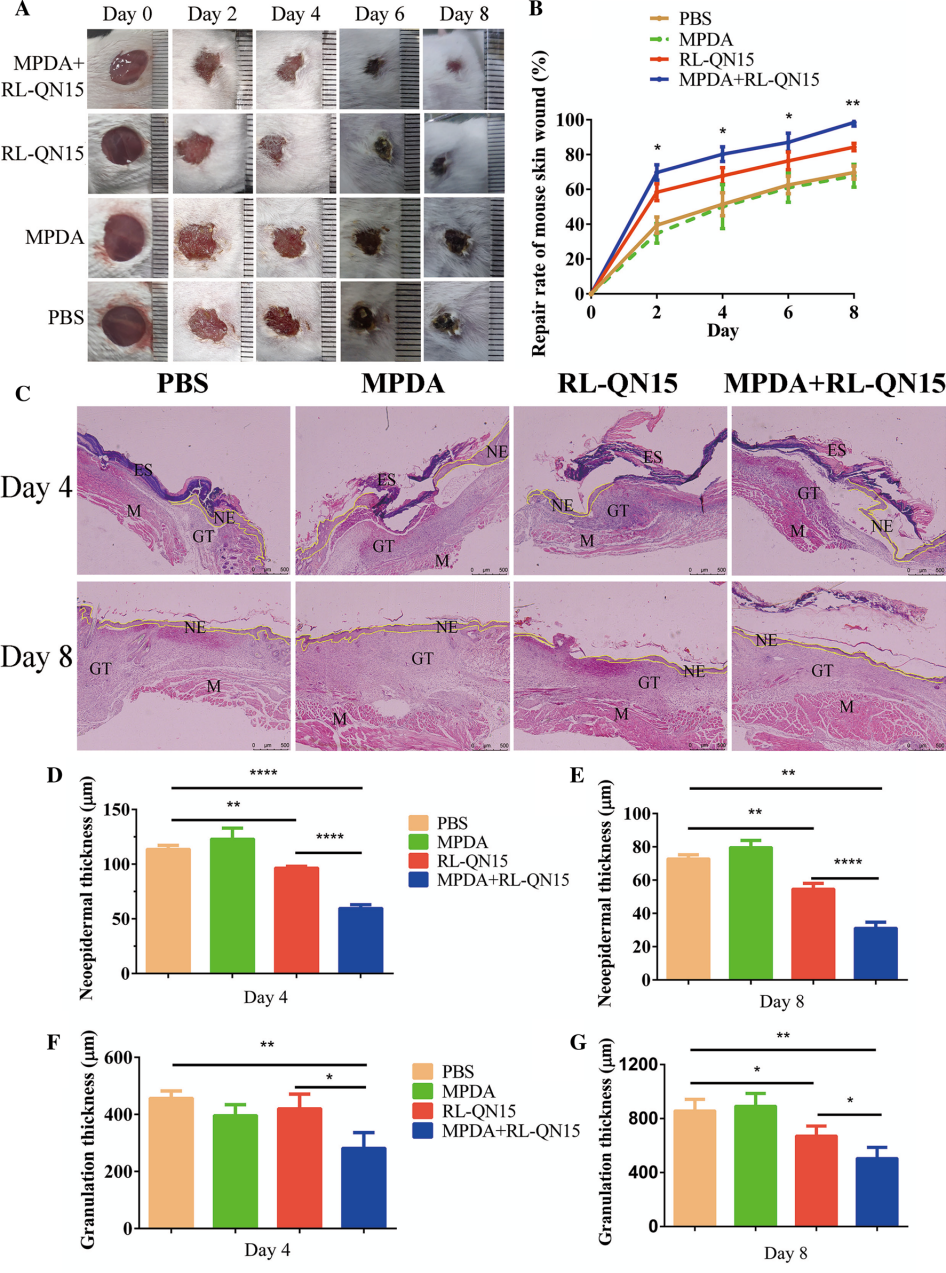
Topical application of nanocomposites of MPDA + RL-QN15 accelerated healing of full-thickness skin wounds in mice. **A** Representative images of skin wounds on days 0, 2, 4, 6, and 8 post-injury in PBS, MPDA, RL-QN15, and nanocomposites of MPDA and RL-QN15-treated groups. MPDA (0.2 mg/mL), RL-QN15 (1 nM), and MPDA (0.2 mg/mL) + RL-QN15 (1 nM) were applied topically twice a day to skin wounds. **B** Quantitative curves of pro-healing activities of PBS, MPDA, RL-QN15, and MPDA + RL-QN15, respectively. **C** Histopathological examination of PBS, MPDA, RL-QN15 and RL-QN15 + PDA-treated full-thickness excisional wounds stained with H&E. NE, neo-epidermal; GT, granulation tissue; ES, eschar; M, muscle; Scale bar: 500 μm. Yellow line indicates neoepithelium. **D**, **E** The quantitative analysis of neo-epidermal thickness on days 4 and 8 post-operative. ** F**, **G** The quantitative analysis of granulation thickness on day 4 and 8 post-operation, respectively. Dates were presented as mean ± S.D. from 9 mice (n = 9). *P < 0.05, **P < 0.01, and ****P < 0.0001 indicated statistical differences between two groups

The full-thickness skin wound model of mice is adopted to preliminarily evaluate whether the load of MPDA could significantly increase the activity of RL-QN15. PBS, PDA, RL-QN15, nanocomposite of MPDA + RL-QN15 was administered topically twice a day to the wounds constructed on the dorsal skin of mice. As shown in Fig. 4A, B, compared with PBS, MPDA (0.2 mg/mL) could not whereas RL-QN15 (1 nM) could significantly promote the skin wound healing. On the eighth post-operative, RL-QN15 increased the healing effect by nearly 20% (**P < 0.01) compared with the control group, showing excellent potential to accelerate wound healing, which was consistent with the data in our previous report [23]. It was worth pointing out that the healing ability of MPDA loaded with lower concentration of RL-QN15 (1 nM) was similar to that of RL-QN15 (50 nM) alone. In other words, MPDA nanoparticles as the carrier of peptides can significantly enhanced the prohealing potency of RL-QN15 by nearly 50 times. Specifically, on the postoperative days 2, 4, 6, and 8, the wound healing rates in the RL-QN15 was 58.35 ± 5.08%, 67.81 ± 5.14%, 76.39 ± 5.56%, 84.3 ± 2.38%, respectively, however, by means of the load of MPDA, healing rate in nanocomposites of MPDA + RLQN15 increased to 69.69 ± 5.1%, 80.24 ± 4.36%, 87.07 ± 5.85%, 98.48 ± 2.46%, respectively, which showed increases of 19.43%, 18.33%,13.98%, 17.14%, respectively. In addition, the repair-promoting activity of nanocomposites of MPDA + RLQN15 was time-dependent (Fig. 4B).

Mice were sacrificed for histological analysis at postoperative days 4 and 8, and the effects of MPDA + RL-QN15 on wound healing in vivo were further investigated. Chronic or non-healing wounds and excessive scarring have consistently been pressing problems in wound healing. On the one hand, once a wound is formed, a great number of fibroblasts and keratinocytes keep proliferating and migrating, which contributes to the generation of granulation tissue and neoepidermal epidermis on the wound surface. Then, on the other hand, excessive proliferation and migration lead to over deposition of extracellular matrix, which is a major cause of scar formation. At the histological level, pathological scarring is characterized by having thick granulation tissue and epidermis. Therefore, the assessment of epidermal re-epithelialization, neoepidermis thickness, and granulation thickness is crucial for wound healing and its healing quality. Here, the H&E images of each group of wounds were quantified by ImageJ software. As shown in Fig. 4C, D, on postoperative day 4, the neoepidermis thickness of mice in PBS, PDA, RL-QN15 groups were 113.29 ± 4.36 μm, 139.84 ± 27.78 μm, 96.68 ± 1.59 μm, respectively, while the neoepidermis thickness of mice in MPDA + RL-QN15 group was only 59.85 ± 3.25 μm (Fig. 4D). On the postoperative day 8, there was no significant difference in the thickness of epidermis between PBS and MPDA groups (72.89 ± 2.35 m vs. 83.74 ± 2.52 μm). The thickness of epidermis in RL-QN15 was about 54.70 ± 3.63 μm, which was significantly lower than that in PBS and PDA groups, but still higher than that in MPDA + RL-QN15 group (31.24 ± 3.87 μm). We also evaluated the granulation tissue thickness, as shown in Fig. 4F, G, on postoperative day 4, there was almost no difference in granulation tissue thickness between the PBS, PDA and RL-QN15 groups, and MPDA + RL-QN15 was lowest among the four groups. On postoperative day 8, there was no significant in granulation thickness between PBS and PDA groups (858.53 ± 96.13 μm vs. 863.71 ± 89.14 μm). Granulation thickness of RL-QN15 was 672.76 ± 74.43 μm, which was significantly lower than that of PBS and PDA groups, but still higher than that of MPDA + RL-QN15 group (506.60 ± 83.62 μm). In a word, after topical administration of nanocomposites of MPDA + RL-QN15, the regeneration and reconstruction of the epidermis and granulation tissue were significantly enhanced.

In previous studies, there are few reports on the combination of nanomaterials and peptides to promote wound healing. One of available references is that KR-12 peptide combined to the fibrous eggshell membrane to promote angiogenesis and hence accelerate skin re-epithelialization [43]. The report on the binding of MPDA particles to peptides as prohealing therapeutics remains unavailable. Our results showed that, by the load by MPDA, the prohealing potency of RL-QN15 against the full-thickness injured wounds in mice were increased by an average of 17.14%. Hence, for the first time, we provided evidence to indicate that MPDA nanoparticles could be used as a peptide-carrier to achieve better pro-healing potency.

### The prohealing potency of RL-QN15 against burn skin wounds was significantly enhanced by the load of MPDA

**Fig. 5:**
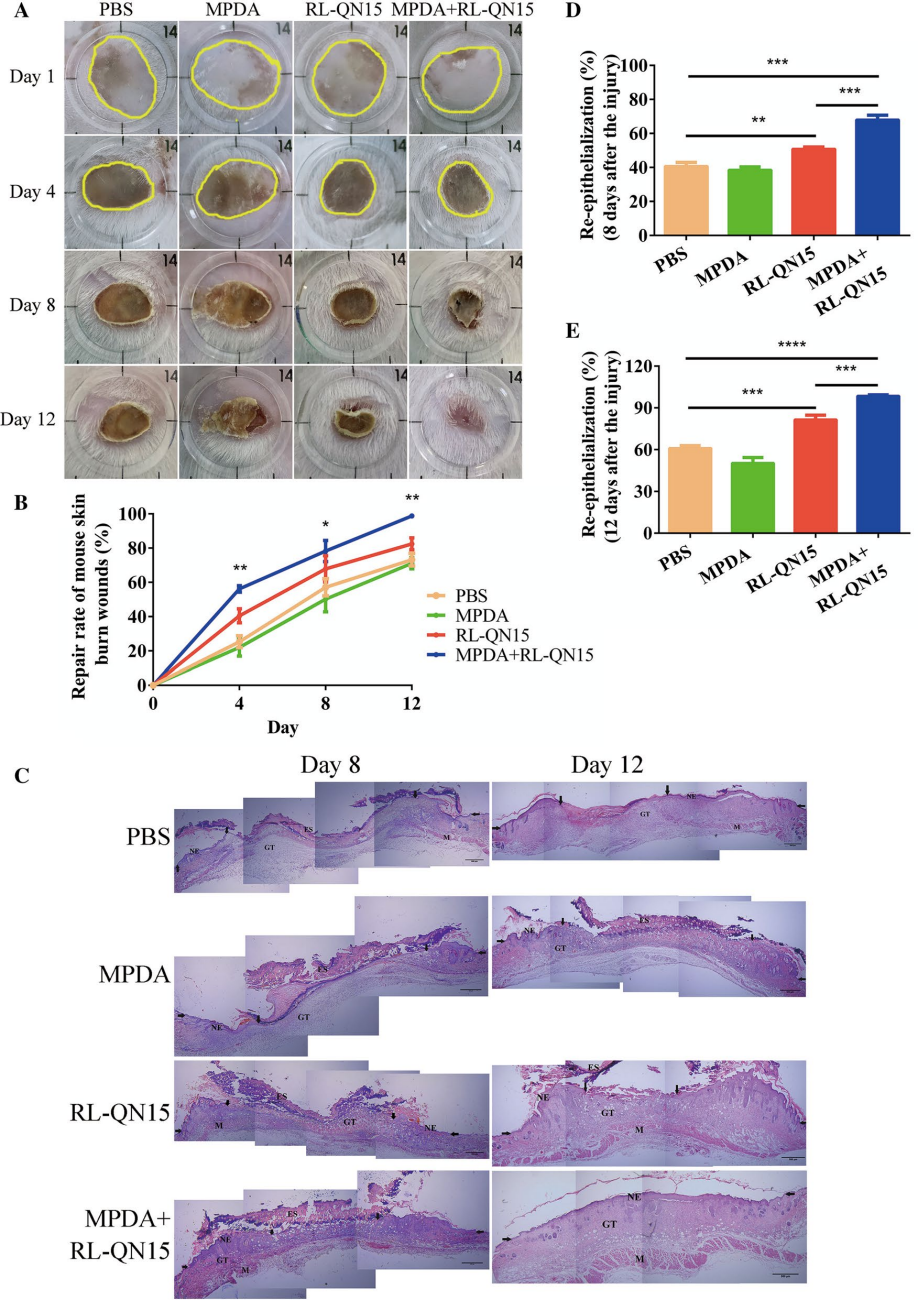
The ability of RL-QN15 against burn wound was obviously improved by the load of MPDA. **A** Representative images of scald wounds on days 0, 4, 8, and 12 post-injury in PBS, MPDA, RL-QN15, and MPDA + RL-QN15 groups. MPDA (0.2 mg/mL), RL-QN15 (1 nM), and nanocomposites of MPDA (0.2 mg/mL) + RL-QN15 (1 nM) were applied topically twice a day to skin wounds. **B** Quantitative curves of pro-healing activities of PBS, MPDA, RL-QN15, and nanocomposites of MPDA + RL-QN15. **C** Representative images of burns sections stained with H&E indicating histomorphological changes in skin wounds on days 8 and 12 post-injury in PBS, MPDA (0.2 mg/mL), RL-QN15 (1 nM), and nanocomposites of MPDA (0.2 mg/mL) + RL-QN15 (1 nM) groups. NE, neo-epidermal; GT, granulation tissue; ES, eschar; M, muscle. **D**, **E** Histological re-epithelialization of epidermis on days 8 and 12 post-injury. Data were presented as mean ± S.D. from 9 mice (n = 9). *P < 0.05, **P < 0.01, and ****P < 0.0001 indicated statistically differences between two groups

Burn is a common trauma in daily life and the clinically available means to treat burn is the application of skin grafting and wound dressing. However, slow wound healing, infection, pain, and hypertrophic scarring continue to remain a major challenge in burn research and management [44]. In our previous research, we demonstrated that RL-QN15 could promote chronic skin wound healing in diabetic mice, but it is unknown whether RL-QN15 could promote burn wound healing. In this study, we successfully established the mice model of deep-II degree burn on the dorsal skins. The macroscopic morphology of the experimentally induced burns was presented in Fig. 5A. After inducing the burns on experimental animals, the local area was characterized by a white eschar, the surface skin layers (epidermis and dermis) were damaged. In the first four days, the wound turned brown due to extravasation of injured cells and an increase in the level of inflammation. Eight days after the burn, the skin formed a scab and gradually fell off. PBS, MPDA, RL-QN15, nanocomposite of MPDA + RL-QN15 was administered twice a day to the burns of mice. As shown in Fig. 5B, MPDA itself could not promote skin wound healing in mice compared with PBS, while RL-QN15 (1 nM) could significantly promote wound healing. On day 12 post-operation, the wound healing rate in the PBS and MPDA groups was only 73.23 ± 4.25% and 71.16 ± 3.73%, respectively, while the wound healing rate in the RL-QN15 group was 82.47 ± 3.53%, which showed that RL-QN15 has the potential to promote the healing of burn wounds in mice. What is worth mentioning is that the prohealing potency 0f RL-QN15 against chronic skin wounds was significantly enhanced by the load of MPDA. On post-operative day 12, the healing rate of MPDA + RL-QN15 was 98.80 ± 0.80%, which was 19.80% higher than that of RL-QN15 (**P < 0.01).

In a parallel experiment, mice were sacrificed for histological analysis at postoperative days 8, and 12, and the effects of nanocomposites of MPDA + RL-QN15 on wound healing *in vivo* were further investigated. Histological analysis indicated that mice topically treated with nanocomposites of MPDA + RL-QN15 displayed prominently accelerating regeneration of neo-epidermis (neo-epithelial tongue) in the wound compared with the PBS, MPDA or RL-QN15 group (Fig. 5C). One of the major systemic damage responses after burn injuries is caused by proinflammatory cytokines released by inflammatory and vascular endothelial cells [45]. During the healing process, the burn wound showed more inflammatory infiltration than the full-thickness wound (Figs. 4C vs. 5C). On the post-injury day 8, all groups were still in the stage of inflammation, while MPDA + RL-QN15-treated group showed the best re-epithelialization and best-formed granulation tissue amongst four groups. On the post-injury day 12, epidermal regeneration and reconstruction of the dermis were complete in the MPDA + RL-QN15 group, which were similar to that in normal mice. Histological evaluation of mice skin tissue sections stained with H&E was also carried out. As illustrated in Fig. 5D, E, during the healing process, the level of re-epithelialization in MPDA + RL-QN15 group was always higher than that in other groups. On postoperative day 12, neo-epithelial tongue was completely covered the whole wounded area in the MPDA + RL-QN15-treated mice (re-epithelialization 98.80 ± 1.15%). In contrast, only little partial neo-epithelial tongue was found in the MPDA (re-epithelialization of 50.33 ± 4.64%) and most partial neo-epithelial tongue in the RL-QN15 (re-epithelialization of 82.47 ± 3.67%). In conclusion, histological analysis revealed that mice treated with MPDA + RL-QN15 showed better granulation tissue contraction, and high re-epithelialization.

This is the first time to prove that RL-QN15 showed pro-healing activity on burns in the dorsal skins of mice. Moreover, the load of MPDA significantly enhanced the prohealing potency of RL-QN15. Although RL-QN15 could promote the healing of full-thickness, burns and diabetic wounds in mice, the difference in skin structure between mice and humans made it impossible to provide scientific means for clinical treatment of wound. Considering the clinical application, we next explored the healing activity of RL-QN15 in porcine full-thickness injury.

### The effect of RL-QN15 against full-thickness dorsal skin wounds in swine was significantly accelerated by the load of MPDA

Due to the panniculus carnosus, healing in these small animals is largely achieved through wound contraction, as opposed to re-epithelialization in humans [46, 47]. Furthermore, the murine epidermis is only 50 μm thick, so it is technically difficult to create partial-thickness wounds [48]. Porcine models have emerged as promising models to study wound healing. An advantage of using swine is that they are anatomically and physiologically similar to humans [49], and have been used to study many other diseases [50–52]. Like humans, they have a relatively thick epi-dermis, distinct rete pegs, dermal papillae, and dense elastic fibers in the dermis [53, 54]. Swine also have sparse hair rather than fur, although the hair is coarser than human hair. Similarities between swine and human skin also make swine an appropriate choice for the construction of cutaneous wound healing animal models.

**Fig. 6:**
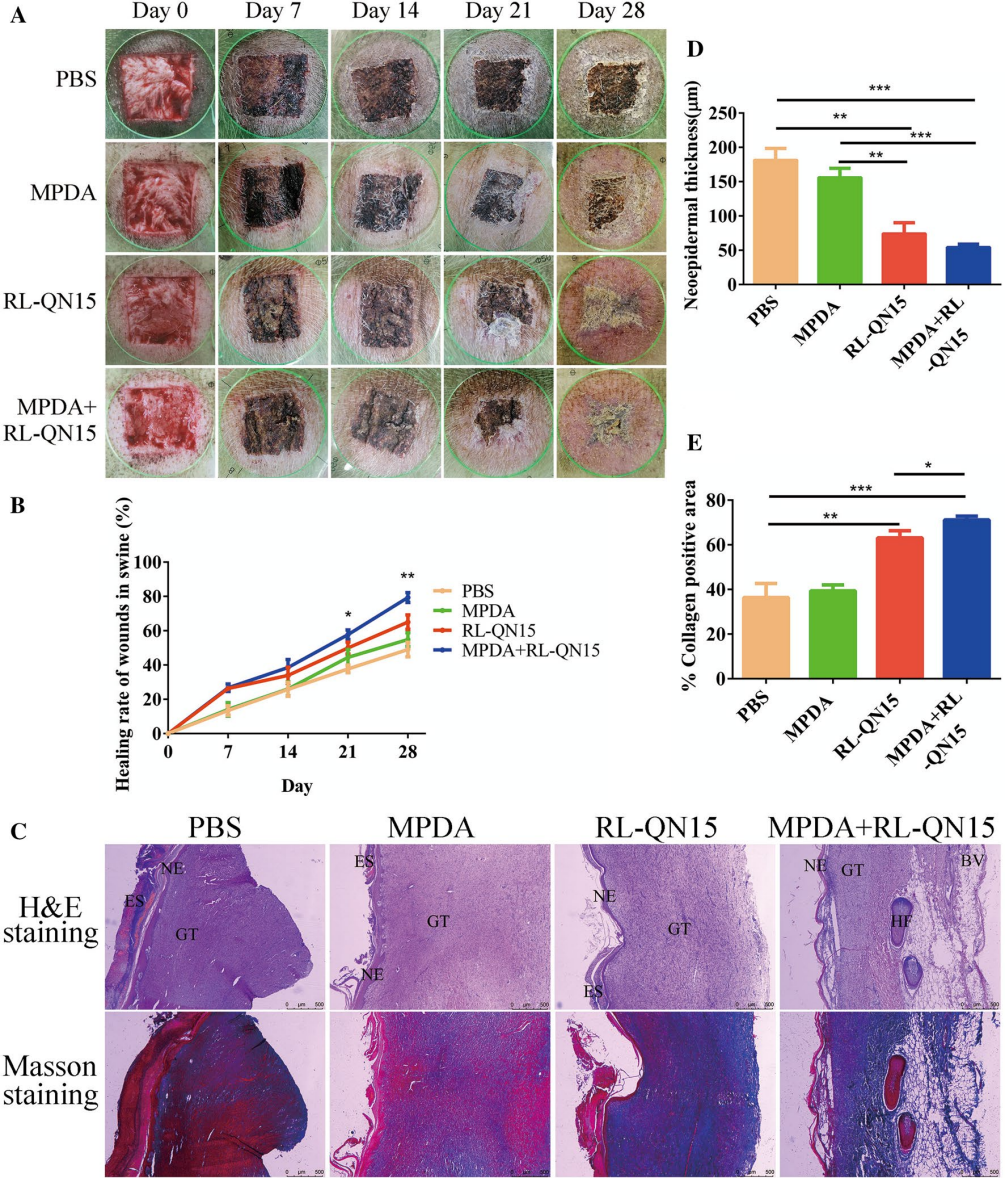
The effect of RL-QN15 against full-thickness dorsal skin wound in swine was enhanced by the load of MPDA. **A** Representative images of full-thickness wounds on days 0, 7, 14, 21, and 28 post-injury in PBS, MPDA, RL-QN15, and nanocomposites of MPDA + RL-QN15 groups. MPDA (0.2 mg/mL), RL-QN15 (250 nM), and MPDA (0.2 mg/mL) + RL-QN15 (250 nM) were applied topically twice a day to the wounds. **B** Quantitative curves of pro-healing activities of PBS, MPDA, RL-QN15, and nanocomposites of MPDA + RL-QN15. **C** Representative images of skin wound sections stained with H&E and Masson’s trichrome staining on days 28 post-injury in PBS, MPDA (0.2 mg/mL), RL-QN15 (250 nM), and nanocomposites of MPDA (0.2 mg/mL) + RL-QN15 (250 nM) groups. NE, neo-epidermal; GT, granulation tissue; ES, eschar; BV, blood vessels; HF, hair follicle. **D** The quantitative analysis of neo-epidermal thickness on day 28 post-operative. **E** Quantification of collagen positive area on day 28 post-operative. Data were presented as mean ± S.D. from 6 wounds (n = 6). *P < 0.05, **P < 0.01, and ****P < 0.0001 indicated statistically differences between two groups

A full-thickness dermal skin wound model was established in swine to assess whether the load of MPDA could significantly increase the prohealing activity of RL-QN15. The full-thickness wounds of the dorsal skins were treated with PBS, PDA, RL-QN15, nanocomposite of MPDA + RL-QN15 twice a day. As shown in Fig. 6A, B, compared with PBS, MPDA could hardly promote wound healing of swine skin, while RL-QN15 significantly promoted wound healing. For example, on day 28 post-operation, the wound healing rate in PBS and MPDA groups was only 49.01 ± 4.48% and 54.85 ± 4.11% (n = 6) respectively, while in RL-QN15 group, the wound healing rate was 65.02 ± 4.70% (n = 6). In particular, the effect of RL-QN15 against full-thickness dorsal skin wounds in swine was significantly accelerated by the load of MPDA. For instance, at day 7 and 14, there was almost no difference between the two groups, however, at postoperative days 21 and 28, the wound healing rates in the nanocomposite of MPDA + RL-QN15 group was 57.67 ± 2.98% and 79.35 ± 3.30% (n = 6), respectively, which showed increases of 15.43% (*P < 0.05), 21.86% (**P < 0.01) compared with RL-QN15 group.

Histological analysis was also carried out on skin sections (stained using H&E and Masson’s trichrome) from swine on day 28 post-operation. New blood vessels and hair follicles appeared in the group treated with MPDA + RL-QN15 rather than RL-QN15 groups (Fig. 6C). In addition, the thickness of new epidermis in RL-QN15 and MPDA + RL-QN15 groups was 74.25 ± 15.89 μm, 54.46 ± 4.33 μm (n = 6) respectively, which was significantly lower than that in PBS group (181.52 ± 17.83 μm) and MPDA group (156.14 ± 14.81 μm) (n = 6) (Fig. 6D). These results show that RL-QN15 not only accelerated wound healing, but also effectively improved the increase in skin thickness, which is consistent with our previous research [23]. In addition, we also quantified the proportion of collagen. There was no significant difference in the proportion of collagen between PBS and MPDA groups (36.39 ± 6.78% vs. 39.38 ± 2.84% (n = 6)) and the proportion of collagen in RL-QN15 was about 63.11 ± 3.22% (n = 6), which was significantly higher than that in PBS and PDA groups, but still lower than that in MPDA + RL-QN15 group (71.13 ± 1.78%) (n = 6) (Fig. 6E). In a word, as a carrier of RL-QN15, MPDA successfully improved the prohealing strength of RL-QN15.

### The construction of nanocomposite of MPDA and prohealing peptide might be a promising option for the development of novel prohealing interventions

**Fig. 7:**
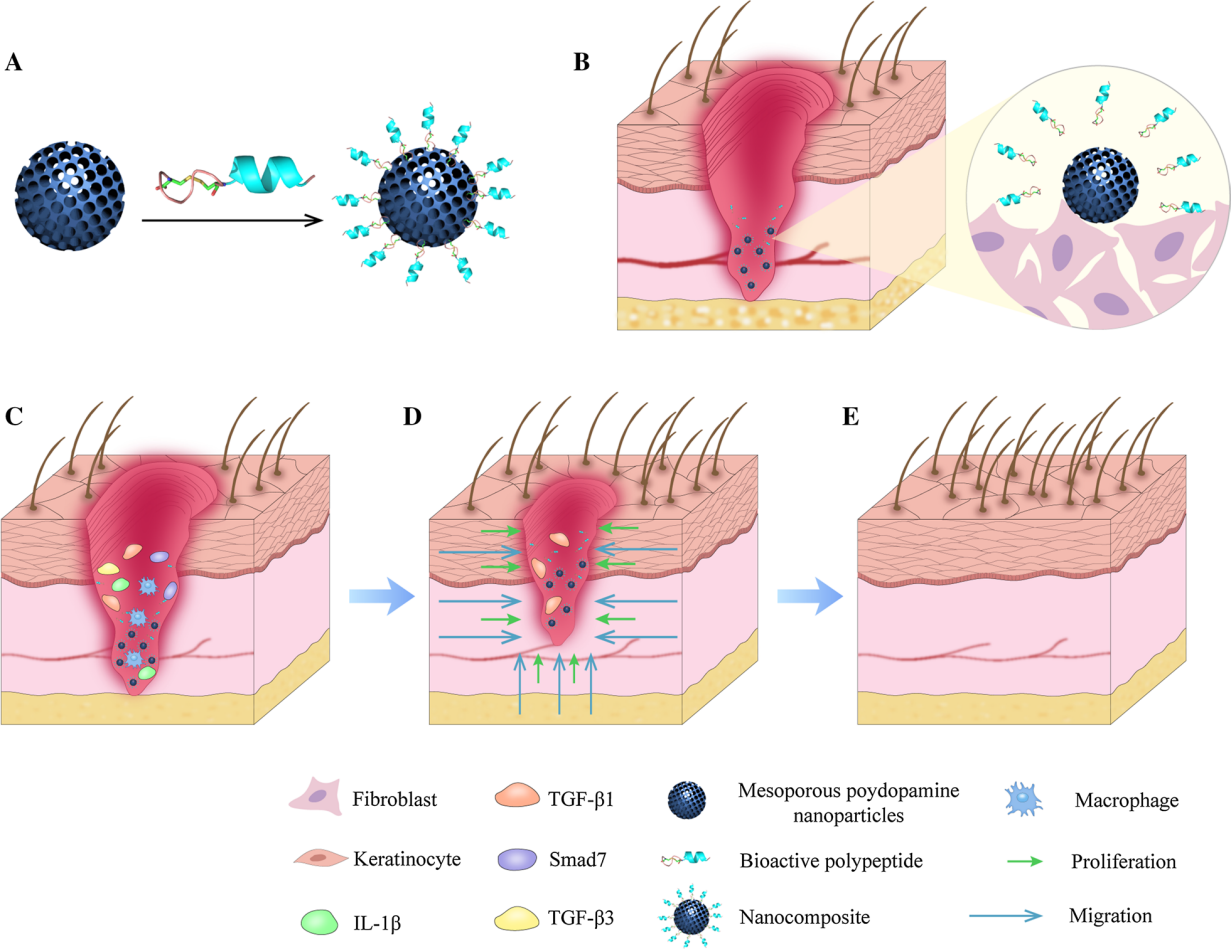
Schematic diagram of MPDA drug delivery system to improve the healing effect of RL-QN15. **A** Successful preparation of nanocomposites of MPDA and RL-QN15. **B** MPDA released RL-QN15 on the surface of wound. **C** RL-QN15 induced the cytokines secretion from macrophages. **D** The migration and proliferation of keratinocytes and fibroblasts induced by RL-QN15. ** E** The wounds treated with nanocomposites of RL-QN15 healed completely

At present, the available intervention for wound repair mainly include various growth factors, small molecular compounds, wound dressings and hydrogels, which are far from meeting the clinical needs, thus the development of novel wound healing therapy is urgently highlighted [55]. In our study, the peptide RL-QN15 secreted from the skin of R. *limnocharis* frogs was loaded onto MPDA particles (Fig. 7A). The MPDA particles as drug delivery system loaded with peptides adhere to the wound surface and release RL-QN15 (Fig. 7B). Then, according to our previous research, during the inflammatory phase, RL-QN15 stimulates inflammatory cells, especially macrophages, to secrete cytokines such as TGF-β1 [23] (Fig. 7C). Which is a multifunctional growth factor that exerts pleiotropic effects on wound healing by regulating cell proliferation and migration, differentiation, ECM production, and immune modulation [56]. Next, the wound enters the proliferative phase. TGF-β1 and RL-QN15 promote the proliferation and migration of skin fibroblasts and keratinocytes, resulting in wound closure (Fig. 7D). Finally, during the tissue remodeling period, the skin in the wound site is fully restored, including primarily restoration of the epidermal and dermal layers with appendages, such as hair follicles (Fig. 7E).

Many nanoparticles have been found to promote wound healing through their intrinsic characteristics (antibacterial, anti-oxidant, promote cell proliferation and migration). For example, cerium oxide nanoparticles accelerate the healing of full-thickness dermal wounds in mice via enhancement of the proliferation and migration of fibroblasts, keratinocytes [57]. In addition, compared to porous beads, some functional nanofiber-based scaffolds and antimicrobial dressings are gradually being investigated and applied as an effective strategy for therapeutic wound healing [58–61]. MPDA nanoparticles showed no healing ability, but improved the healing effect of RL-QN15. On the one hand, MPDA nanospheres prolonged the effective concentration of RL-QN15 on the wound surface by continuously releasing RL-QN15. On the other hand, the MPDA microsphere shell protected RL-QN15 from modification by endogenous (e.g., tyrosinase, elastase, metalloproteinase) and exogenous (e.g., produced by colonized microorganisms) enzymes, thus enhancing the therapeutic effect. Therefore, the construction of nanocomposite of MPDA and prohealing peptide might be an option for the development of novel prohealing interventions.

## Conclusions

In the current study, the MPDA nanoparticles and nanocomposites of MPDA and RL-QN15 were successfully prepared. Our results indicated that, compared with RL-QN15 alone, nanocomposites of MPDA and RL-QN15 showed significant increases in the prohealing potency in the full-thickness injured wounds (mice and swine) and burn wounds (mice). Based on our knowledge, this is the first research to report that the load of MPDA nanoparticles could significantly increase the prohealing potency of peptide and hence highlighted the nanocomposites of MPDA and RL-QN15 as a promising intervention for the acceleration of skin wounds.

## Supplementary information

**Additional file 1: Figure S1**. Infrared spectra between 1800cm-1-400cm-1. **Table S1**. Surface area, pore volume and pore diameter of MPDA nanoparticles.

## Data Availability

The datasets used and/or analyzed during the current study are available from the corresponding author on reasonable request.
