# Supplementary material for: Mesoporous polydopamine nanoparticles carrying peptide RL-QN15 show potential for skin wound therapy
Source: J Nanobiotechnology. 2021 Oct 9;19:309. doi: 10.1186/s12951-021-01051-8 (PMC8501717; doi:10.1186/s12951-021-01051-8)
Supplement: Supplementary file 1 — Additional file 1: Figure S1. Infrared spectra between 1800cm-1-400cm-1. Table S1. Surface area, pore volume and pore diameter of MPDA nanoparticles. [file 12951_2021_1051_MOESM1_ESM.docx]

**Mesoporous Polydopamine Nanoparticles Carrying Peptide RL-QN15 Show Potential for Skin Wound Therapy**

Pan Qin^4,$^, Yi Meng^1,$^, Ying Yang^5,$^, Xinyu Gou^2,$^, Naixin Liu^1^, Saige Yin^1^, Yan Hu^1^, Huiling Sun^1^, Zhe Fu^1^, Yinlei Wang^1^ , Xiaojie Li^4^, Jing Tang^4^, Ying Wang^3,*^, Ziwei Deng^2,*^, Xinwang Yang^1,*^

1. Department of Anatomy and Histology and Embryology, Faculty of Basic Medical Science, Kunming Medical University, Kunming, Yunnan, 650500, China.

2. Key Laboratory of Applied Surface and Colloid Chemistry, National Ministry of Education, Shaanxi Key Laboratory for Advanced Energy Devices, Shaanxi Engineering Lab for Advanced Energy Technology, School of Materials Science and Engineering, Shaanxi Normal University, Xi’an, Shanxi, 710119, China.

3. Key Laboratory of Chemistry in Ethnic Medicine Resource, State Ethnic Affairs Commission & Ministry of Education, School of Ethno-Medicine and Ethno-Pharmacy, Yunnan Minzu University, Kunming, Yunnan, 650504, China.

4. Department of Biochemistry and Molecular Biology, Faculty of Basic Medical Science, Kunming Medical University, Kunming, Yunnan, 650500, China.

5. Department of Endocrinology and Metabolism, Second People’s Hospital of Yunnan Province & Affiliated Hospital of Yunnan University, Kunming, Yunnan, 650021, China.

^$^These authors contributed equally to this work.

^*^ Corresponding authors:

Prof. Xinwang Yang, Faculty of Basic Medical Science, Kunming Medical University, Email: yangxinwanghp@163.com

Prof. Ziwei Deng, School of Materials Science and Engineering, Shaanxi Normal University. E-mail: zwdeng@snnu.edu.cn

Prof. Ying Wang, Key Laboratory of Chemistry in Ethnic Medicine Resource, State Ethnic Affairs Commission & Ministry of Education, School of Ethno-Medicine and Ethno-Pharmacy, Yunnan Minzu University, Email:[wangying_814@163.com](mailto:wangying_814@163.com)


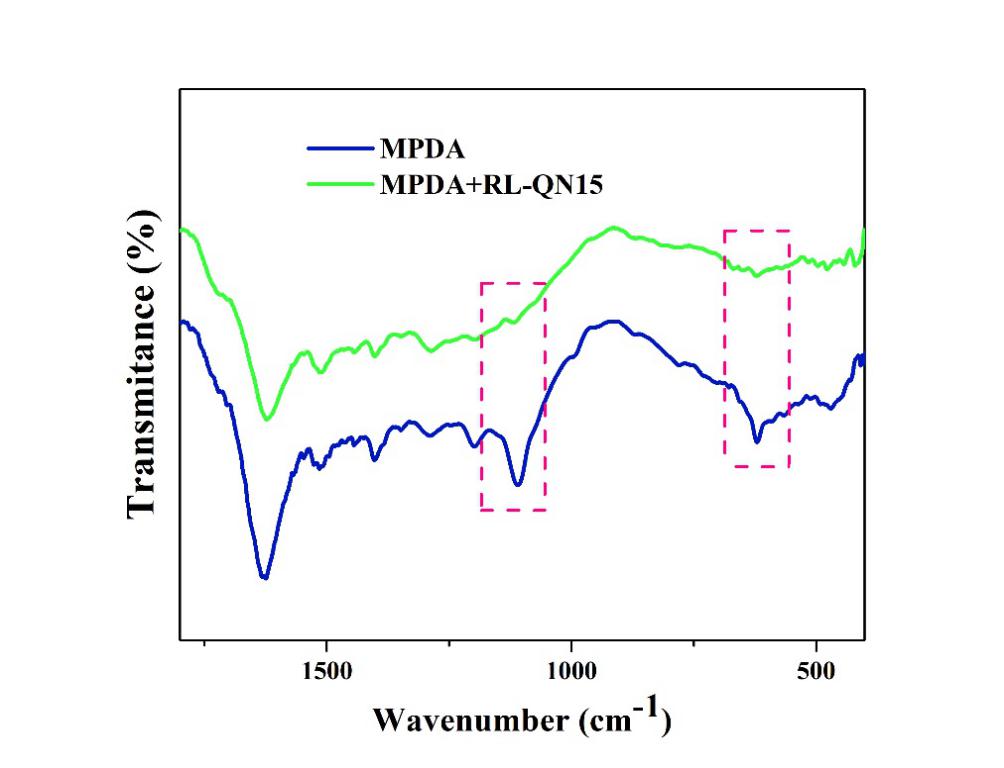


**Figure S1: Infrared spectra between 1800cm^-1^-400cm^-1^.**

**Table S1. Surface area, pore volume and pore diameter of MPDA nanoparticles.**

| BET Surface area(m^2^/g) | pore volume (cm^3^/g) | pore diameter (nm) |
| --- | --- | --- |
| 21.5475 | 0.119326 | 21.3857 |
